# A study on the pathways through which pet ownership influences fertility intentions

**DOI:** 10.3389/fpsyg.2026.1822148

**Published:** 2026-09-30

**Authors:** Caifeng Tan, Wenfei Qi, Yuxin Zhao, Yuhang Xiao

**Affiliations:** 1 School of Economics, Hunan Agricultural University, Changsha, China; 2 College of Economics & Management, Huazhong Agricultural University, Wuhan, China; 3 Department of Agricultural and Resource Economics, North Carolina State University, Raleigh, NC, United States

**Keywords:** emotional value, fertility intentions, fertility motivation, intrinsic needs, pet ownership behavior, self-determination theory

## Abstract

Against the backdrop of China’s low fertility rate, examining the impact of pet ownership on fertility intentions and its underlying mechanisms holds significant importance for enriching research on factors influencing fertility decisions and for understanding the complex relationship between modern lifestyles and reproductive choices. This study, grounded in self-determination theory, collected 551 valid samples through questionnaire surveys and employed structural equation modeling for analysis. Findings reveal that pet ownership not only positively associated with fertility intentions but also indirectly affects them through mediating variables such as fertility motivation, intrinsic needs, and emotional value. Pet ownership appears to individuals’ positive expectations toward parenthood by providing opportunities for daily care, emotional companionship, and responsibility-taking; simultaneously, the nurturing experience and emotional bonds cultivated through pet ownership activate individuals’ intrinsic motivation toward the role of “parenthood.” This may fulfill fundamental psychological needs such as autonomy, competence, and relatedness, thereby strengthening reproductive motivation. The positive emotional experiences and heightened confidence in family life derived from pet ownership may significantly reduce sensitivity to reproductive pressures, indirectly supporting the willingness to have children. This study provides theoretical support and empirical evidence for understanding the relationship between pet ownership and fertility intentions, as well as for formulating relevant policies.

## 1 Introduction

In recent years, profound changes in China’s demographic structure have made low fertility a pressing social issue. According to National Bureau of Statistics data, China’s total fertility rate has fallen to a historic low, far below the replacement level required for population stability (Feng and Cui, 2022). This phenomenon not only challenges long-term balanced population development but also profoundly impacts socioeconomic sustainability (Zhang et al., 2021). To address this challenge, the Chinese government has introduced a series of policy measures in recent years to enhance fertility intentions and birth rates. The “three-child policy” formally implemented in 2021 aims to encourage families to have more children by relaxing birth restrictions. However, relaxing birth restrictions alone is unlikely to fundamentally resolve the issue of low fertility rates. In 2023, the National Health Commission and other departments further issued the “Guiding Opinions on Further Improving and Implementing Active Fertility Support Measures”, explicitly proposing to reduce the costs of childbirth, childcare, and education for families through multiple channels, including economic support, childcare services, housing security, and other measures. These policies demonstrate the government’s high priority and multidimensional support for fertility issues, reflecting that fertility intentions are influenced not only by policy factors but also closely related to micro-level factors such as individual lifestyles and psychological needs.

Traditional research has predominantly focused on the impact of macro-level policy factors and social structures on fertility intentions (Cao et al., 2025). However, with societal development and the awakening of individual consciousness, the role of micro-level lifestyle choices and behavioral preferences in shaping fertility intentions has become increasingly prominent. Among these, pet ownership—as an increasingly prevalent lifestyle choice (Liu and Yang, 2026) —has not received sufficient attention regarding its potential association with on individual fertility intentions. In modern society, pets have evolved from mere companions to full-fledged family members, even symbolizing emotional support and life responsibilities (Li and Shao, 2025). Moreover, accelerated urbanization and faster-paced lifestyles subject modern youth to mounting pressures and psychological challenges, including rising housing costs, intense workplace competition, and strained social relationships (Liu and Liu, 2025). Against this backdrop, pet ownership serves not only as a means of fulfilling emotional needs but also as a way to cope with life’s pressures, cultivate a sense of responsibility, and foster family values. However, research on how pet ownership relates to fertility decision-making remains limited. Therefore, this paper attempts to explore, from the perspective of self-determination theory (SDT), how pet ownership relates to individuals’ psychological needs, motivations, and emotional value, thereby potentially affecting their fertility intentions. Through systematic theoretical analysis and empirical research, this study aims to provide new perspectives and theoretical support for understanding the complex relationship between modern lifestyles and fertility intentions. Furthermore, this study seeks to extend the application boundary of SDT in the field of fertility decision-making research. While SDT has been widely applied in domains such as education, health behavior, and work motivation, its explanatory power in reproductive contexts remains underexplored. By demonstrating how voluntary caregiving behavior (pet ownership) activates autonomous motivation for parenthood through the satisfaction of basic psychological needs, we expand SDT’s traditional application from individual goal pursuit to life-transition decisions involving family formation.

## 2 Literature review and hypothesis formulation

In recent years, as China’s total fertility rate continues to decline, the fertility intentions of the youth population have gradually become a core topic of academic attention (Li et al., 2025; Zhao et al., 2023). Compared to earlier research focused on policy factors, scholars have increasingly turned to micro-level mechanisms such as social psychology, behavioral motivations, and life contexts to explore the underlying logic influencing fertility intentions (Liu and He, 2024). Lifestyle behaviors, as external manifestations of individual values and emotional needs, are increasingly recognized as a crucial entry point for understanding reproductive decisions (Zhang and Chen, 2025). Pet ownership, as a quintessential lifestyle choice (Xin, 2023), not only reflects an individual’s identification with intimacy, responsibility, ethics, and family life, but may also subtly relate to their acceptance of parenting roles and psychological expectations regarding childbearing.

Although existing research has extensively examined the impact of structural factors such as housing stress (Zhang and Zhu, 2022), workplace anxiety (Li and Lan, 2017), and gender inequality on fertility intentions, systematic theoretical explanations and empirical analyses remain lacking regarding how pet ownership behavior is embedded within the fertility decision-making process. This paper attempts to use SDT as an analytical framework to explore how pet ownership relates to young adults’ fertility intentions by potentially stimulating intrinsic motivation, satisfying psychological needs, and providing emotional value. Specifically, the literature review and hypothesis development will unfold across the following three dimensions.

### 2.1 Self-determination theory: an overview

Self-determination theory is a macro-theory of human motivation and personality that concerns people’s inherent growth tendencies and innate psychological needs (Ryan and Decl, 2000). Central to SDT is the distinction between intrinsic motivation, where behaviors are performed out of interest and enjoyment, and extrinsic motivation, where behaviors are performed to obtain separable outcomes. The theory posits that three innate psychological needs—autonomy (the need to experience choice and volition), competence (the need to feel effective in one’s activities), and relatedness (the need to feel connected to and cared for by others)—are essential for fostering intrinsic motivation, self-regulation, and well-being. When these needs are satisfied, individuals are more likely to internalize external regulations and engage in self-determined behaviors aligned with their personal values. In the context of fertility decisions, SDT suggests that reproductive choices may be shaped not only by external pressures (e.g., social expectations, policy incentives) but also by the degree to which individuals experience intrinsic motivation and psychological need satisfaction in caregiving contexts. This framework provides a useful lens for understanding how pet ownership, as a voluntary caregiving behavior, may relate to fertility intentions through motivational and emotional pathways.

### 2.2 Pet ownership and fertility intentions

SDT posits that when an action aligns strongly with an individual’s intrinsic values or life goals, it may foster positive expectations for future related behaviors. Pet ownership not only reflects an individual’s emphasis on intimacy, responsibility, and emotional responsiveness but also indicates their identification with and aspiration toward a “family life” model (Volsche and Gray, 2016). Research indicates that pet owners significantly outperform non-owners in family values, emotional expression, and sense of responsibility, and are more likely to view caring for others as a meaningful life practice. This positive experience and psychological identification with parenting roles may directly relate to their acceptance and anticipation of the parenthood life stage (Marks et al., 1994). Therefore, this paper proposes:

*H1*: Pet ownership behavior is positively associated with fertility intentions.

### 2.3 Pet-owning behavior, procreative motivation, intrinsic needs, and fertility intentions

Within the SDT framework, motivation is viewed as an individual’s intrinsic inclination toward goal-directed behavior, serving as the starting point for self-determined actions (Liu, 2012). Unlike the external drives emphasized by traditional behaviorism, SDT stresses that motivation must originate from an individual’s internal identification with the meaning of the behavior—that is, “I want to do it” rather than “I should do it.” Pet ownership behavior activates the intrinsic motivation for the role of “becoming a parent” through the “care-response-attachment” interaction structure (Zhen and Wang, 2023; Sun and Men, 2024). This allows individuals to repeatedly experience the meaning, responsibility, and value of nurturing in a context free from external pressure.

Existing research indicates that surrogate parenting behaviors effectively enhance individuals’ acceptance and identification with the parenting role. Particularly when receiving positive feedback from caregiving, individuals are more likely to transfer the mental schema of “caring for pets” to future scenarios of “caring for children.” Behaviors such as “daily care,” “health monitoring,” and “emotional responsiveness” during pet ownership exhibit high similarity to parenting behaviors (Pan and Huang, 2026). This behavioral simulation mechanism provides a practical foundation for generating fertility motivation. Therefore, this paper proposes:

*H2*: Pet ownership behaviors influence fertility motivation, thereby enhancing reproductive willingness.

Furthermore, SDT posits that motivation formation and maintenance depend on fulfilling three fundamental psychological needs: autonomy, competence, and relatedness (Zhang and Cao, 2026; Fang et al., 2018). Pet ownership behavior supports all three dimensions: individuals autonomously choose whether and how to raise pets, enhancing their sense of control over life (autonomy); through feeding, cleaning, training, and illness care, they gain the experiential sense of “I can care for others” (competence); pets provide stable, unconditional, non-judgmental emotional responses, fulfilling individuals’ needs for intimacy and emotional belonging (Chen and Jiang, 2020) (relational). When these three needs are met, individuals are more likely to transfer caregiving experience to parenting preparation, thereby strengthening their internal identification with and behavioral motivation for the parenting role. Therefore, this paper proposes:

*H3*: Pet ownership is associated with fertility motivation by enhancing intrinsic needs, thereby increasing the desire to have children.

### 2.4 Pet ownership behavior, emotional value, and fertility intentions

SDT emphasizes that positive emotions are not only outcomes of self-determined behavior but also crucial mechanisms reinforcing future behavioral expectations. Emotional value refers to the affective benefits individuals derive from a behavior, such as security, companionship, happiness, and meaning (Duan, 2024; Xiao, 2024). Pet ownership is associated with enhanced life satisfaction and emotional stability by providing consistent emotional responses, nonverbal companionship, and daily interactions (Zhao and Zhang, 2025a). This emotional nourishment not only alleviates the loneliness and existential anxiety prevalent among modern youth (Feng et al., 2024) but may also translate into positive expectations for family life, reducing sensitivity to parenting pressures.

Research indicates that individuals with more positive emotional experiences (Li, 2022) tend to view parenting as an investment in happiness rather than a burden. This is particularly true when individuals experience an emotional cycle of “being needed,” “being responded to,” and “being accompanied” through their pets (Yang and Li, 2022), they are more likely to envision parent–child relationships as sustainable, rewarding emotional bonds (Yang and Song, 2025), thereby strengthening emotional commitment to procreation. Furthermore, emotional value can alleviate young people’s anxiety about parenting pressures (Zhao and Zhang, 2025b), lowering psychological barriers to “childbearing fear.” Therefore, this paper proposes:

*H4*: Pet ownership enhances emotional well-being, thereby increasing the desire to have children.

## 3 Materials and methods

### 3.1 Transparency and openness

This study was conducted in strict accordance with ethical research standards. Although formal ethics committee approval was not required by our institution for this low-risk, anonymous questionnaire study, the research was conducted in full compliance with established ethical guidelines for survey-based research. Prior to participation, all respondents were presented with an informed consent statement explaining the purpose of the study, the voluntary nature of their participation, and their right to withdraw at any time. By completing and submitting the questionnaire, respondents provided their informed consent. The survey did not collect any personally identifiable information, ensuring complete anonymity of all responses. Data were stored securely and used exclusively for this academic research. No monetary or material incentives were offered to respondents; participation was entirely voluntary. The absence of incentives was intended to minimize participation bias and ensure that responses reflected genuine attitudes rather than extrinsic motivation. The data were collected through the WenJuanXing platform^1^, a nationally recognized professional survey platform in China with ISO 27001 information security certification. Wenjuanxing represents one of China’s largest and most extensively utilized online survey platforms, primarily catering to the data collection needs of academic researchers, enterprises, and governmental organizations. Functionally, it can be considered analogous to Qualtrics or SurveyMonkey, which are widely adopted in Western countries, including the United States. Presently, it has amassed over 6 million registered users, spanning various geographical regions and demographic strata across China. The survey was administered in October 2025, covering 28 provinces, municipalities, and autonomous regions across China, yielding 551 valid responses after rigorous quality control procedures. The sampling method employed was a nonprobability-based convenience sample, in which individuals using social media platforms (primarily WeChat) were targeted and invited to participate through targeted group sharing. This approach is common in Chinese social science research due to its cost-effectiveness and broad reach, though it inherently limits the representativeness of the sample. Due to the sensitive nature of the data, which includes personal fertility intentions, family circumstances, and demographic information, the dataset is not publicly available. The data are securely stored and accessible only to the authorized research team, in compliance with institutional ethical guidelines and privacy protection regulations.

### 3.2 Measures and instruments

This study employed a self-designed questionnaire titled “Survey Questionnaire on the Relationship Between Pet Ownership and Fertility Intentions” to collect data. The questionnaire was developed specifically for this research based on the theoretical framework of SDT and an extensive review of existing literature on pet ownership behavior, fertility intentions, and psychological needs. No previously validated instrument was available that simultaneously captured all five constructs of interest; therefore, the research team constructed original items tailored to the study’s objectives.

Several limitations should be acknowledged. First, our study relies on self-reported measures of fertility intentions rather than actual fertility behaviors. While behavioral intention is theoretically the strongest predictor of actual behavior, hypothetical self-reports may not perfectly correspond to real-world decisions. Future research should employ longitudinal designs to track whether pet ownership-related intentions translate into actual childbearing. Second, the inclusion of non-pet owners required adapting questionnaire items to hypothetical scenarios, which may introduce hypothetical bias. Although we conducted robustness checks to mitigate this concern and hypothetical scenarios are the standard ethical approach in fertility intention research, we encourage replication studies using alternative methodologies.

For respondents who did not currently own pets, pet-specific questions were answered based on their hypothetical attitudes or past experiences. Specifically, non-pet owners were instructed to respond according to their general perceptions of pet ownership or previous pet-keeping experiences (if any). For example, when asked “I proactively spend time with my pet daily,” non-pet owners were asked to indicate their likely behavior if they owned a pet, based on their attitudes toward pet companionship. If they had never owned a pet, they were asked to respond based on their general disposition toward caregiving behaviors. This approach allowed all respondents to complete the full questionnaire while maintaining conceptual consistency in measuring pet ownership orientation as a latent disposition rather than purely behavioral frequency.

The questionnaire comprises five sections, each corresponding to a key latent variable in the research model. All items were measured using a 5-point Likert scale ranging from 1 (strongly disagree) to 5 (strongly agree), with higher scores indicating stronger endorsement of the respective construct. This consistent scaling approach facilitates comparability across dimensions and aligns with established practices in social science survey research. All multi-item constructs were measured using 5-point Likert scales ranging from 1 (‘strongly disagree’) to 5 (‘strongly agree’). While this format is widely used in survey-based social science research, we acknowledge that unidirectional Likert items may be susceptible to acquiescence response bias—the tendency of respondents to systematically endorse agreement responses regardless of item content. To assess the potential influence of this bias on our findings, we conducted two post-hoc diagnostic procedures. First, we performed Harman’s single-factor test; the first unrotated factor accounted for 20.95% of the total variance, below the 40% threshold commonly used to indicate significant common method variance. Second, we re-estimated our structural equation model with a latent common method factor on which all indicators were allowed to load. The core mediation pathways remained significant after controlling for this method factor, suggesting that acquiescence bias does not substantially threaten the validity of our conclusions:

1. Pet ownership behavior: This section assesses respondents’ pet ownership status, the number of pets owned, the pet ownership atmosphere within their social circle, preferred pet types, and the level of emotional and behavioral investment in pets. For example, one item asks, “Do you currently own a pet?” to establish the foundational ownership status.
2. Motivation for childbearing: In accordance with SDT, this section specifically measures intrinsic motivation (including identity regulation, integration regulation and intrinsic motivation), that is, an individual’s desire to have children based on personal values and intrinsic interests. The questions are designed to focus on self-identity-based motivations for parenthood, reflecting an individual’s sense of meaning and value alignment. For example: “Caring for a pet has deepened my personal appreciation of the unique meaning and fulfillment that comes from nurturing life, and has strengthened my intrinsic desire to experience parenthood” (identity regulation); “I look forward to establishing a meaningful parent-child relationship that enriches my life journey, rather than merely seeking practical support in the future” (integration regulation); ‘When I imagine myself becoming a parent in the future, I feel a genuine sense of personal fulfillment and joy’ (intrinsic motivation). It should be clarified that ‘motivation for childbearing’ in this study does not refer to pure intrinsic motivation in the strict sense of SDT (i.e., where the behavior itself serves as the reward), but rather to a motivational state in which the desire for childbearing has been deeply integrated with personal values and has become a goal of self-harmony, as distinct from controlled motivation driven by external pressures.
3. Inner needs: Aligned with SDT’s three basic psychological needs, this construct measures how pet ownership may satisfy: (a) autonomy—the volitional choice in pet selection, care routines, and lifestyle adjustments without external coercion (e.g., “I autonomously decide how to care for my pet, which enhances my sense of control over life”); (b) competence—the mastery experiences in feeding, health management, and problem-solving that build efficacy beliefs (e.g., “Through caring for my pet, I have gained confidence in my ability to nurture living beings”); and (c) relatedness—the emotional bonds and unconditional responses that fulfill belongingness needs (e.g., “My pet provides me with stable emotional companionship, making me feel needed and connected”). The fulfillment of these needs is hypothesized to activate intrinsic motivation toward parenthood by demonstrating that caregiving can be a self-determined, efficacious, and relationally rewarding experience.
4. Emotional value: This section evaluates the positive emotional experiences derived from pet ownership and their projection onto family life expectations. Items are carefully designed to measure emotional benefits from pet interactions (e.g., “Interacting with my pet makes me feel warm and happy”) rather than direct fertility intentions, thereby avoiding conceptual overlap with the outcome variable. Emotional transference items assess whether these positive experiences extend to anticipated parent–child relationships (e.g., “The warm moments I share with my pet make me look forward to similar experiences with a future child”), distinguishing between current emotional states and future-oriented desires.
5. Fertility intention: This section measures respondents’ reproductive planning, willingness to assume parenting responsibilities, and emotional expectations toward parenthood. For example, one item asks, “I plan to have children in the future,” representing the core behavioral intention dimension.

### 3.3 Sample procedures

Data for this study originated from the “Survey Questionnaire on the Relationship Between Pet Ownership and Fertility Intentions” distributed via the WenJuanXing platform in October 2025. The sample covers a broad age spectrum, ranging from 18 to over 46 years old, with the largest proportion being the reproductive-age cohort (18–35 years, accounting for 69.1%), while also including middle-aged and older participants (36+, 30.9%) to reflect the real-world diversity of pet owners in China, covering 28 provinces, municipalities, and autonomous regions including Hunan, Guangdong, Jiangsu, Sichuan, Shandong, Zhejiang, Chongqing, Xinjiang, Guangxi, Jiangxi, Fujian, Anhui, Hubei, Henan, Hebei, Liaoning, Guizhou, Yunnan, Shaanxi, Shanxi, Gansu, Shanghai, Beijing, Tianjin, Tibet, Heilongjiang, Jilin, and Hainan. The sample covers diversity across urban tiers.

The questionnaire was disseminated via social platforms such as WeChat. A total of 564 responses were collected. After excluding invalid questionnaires—those completed in excessively short time (<120 s), failing verification questions (e.g., failing to answer “Disagree” for Question 21 as instructed), or exhibiting highly consistent or patterned responses—551 valid questionnaires were obtained, yielding a valid completion rate of 97.69%. We note that this proportion (97.69%) represents the rate of valid questionnaires among all submitted responses, not a traditional response rate. As our sample was drawn through a nonprobability-based convenience sampling approach (targeted distribution via an online panel), the total population of potential respondents is unknown, and therefore a traditional response rate—defined as the number of completed surveys divided by the number of eligible individuals contacted—cannot be calculated. This is consistent with standard practices in survey methodology for nonprobability samples, where the focus is on sample quality and data validity rather than response rate calculations. To ensure data quality, we implemented rigorous screening procedures (e.g., attention checks, response time filters, and logical consistency checks) rather than relying on response rate as a proxy for representativeness. The sample size met the requirements for structural equation modeling (SEM) analysis (sample size/number of observed variables ≥ 10:1) (Du, 2009), thereby fulfilling the conditions for subsequent empirical analysis.

Following best practices in survey research for convenience samples, we do not report a traditional response rate. Instead, we report the proportion of valid responses among all submissions as an indicator of data quality control. This approach aligns with the recommendation that for nonprobability samples, researchers should prioritize transparency about sample recruitment, screening criteria, and data cleaning procedures over response rate calculations.

Our reliance on a convenience sample via an online panel limits the generalizability of our findings. A probability-based sampling approach would be needed to calculate a true response rate and to ensure population representativeness. However, given the exploratory nature of our study and the feasibility constraints of reaching pet-owning populations, the convenience sampling approach was a pragmatic choice, and we have been transparent about its limitations.

### 3.4 Indicator selection

#### 3.4.1 Outcome variables

This latent variable was constructed to capture the multidimensional nature of reproductive planning, encompassing behavioral intention (“I plan to have children in the future”), cognitive acceptance of responsibility (“I am willing to take on the responsibility of raising a child”), emotional anticipation (“I look forward to having my own child”), and willingness toward multiple children (“I believe having two children contributes to family completeness” and “I look forward to the companionship and growth between siblings”). These indicators collectively reflect the cognitive, emotional, and behavioral dimensions of fertility intentions.

#### 3.4.2 Explanatory variables

Pet Ownership Behavior: Pet ownership behavior was assessed through three core dimensions: baseline status, type preferences, and emotional investment. The baseline status includes indicators for current ownership, the number of pets, and the social circle’s pet-owning environment, reflecting how the external atmosphere influences individual perceptions. Pet type preferences were measured to analyze how pet-owning heterogeneity and varying levels of care difficulty impact fertility intentions. Finally, pet investment and emotional bonding were captured through indicators of behavioral commitment, daily time investment, and the perceived significance of pet ownership. Collectively, these dimensions reflect the strength of the human-animal bond and serve as the primary explanatory variables for subsequent analyses.

#### 3.4.3 Outcome variables

Drawing on self-determination theory and the questionnaire’s measurement of the pet ownership-fertility linkage mechanism, three mediating variables—fertility motivation, intrinsic needs, and emotional value—were selected to analyze the intermediate transmission mechanism through which pet ownership influences fertility intentions.

##### 3.4.3.1 Motivation for childbearing

Motivation for Childbearing: Drawing on SDT’s conceptualization of autonomous motivation, this construct captures the internally driven psychological readiness for parenthood that emerges from self-endorsed values, personal growth aspirations, and genuine identification with the caregiving role. The measurement items assess self-endorsed reproductive desires that reflect personal meaning-making and value alignment. Nurturing fulfillment recognition captures identified regulation—the recognition that parenthood is personally valuable. Relational aspiration distinguishes autonomous relational desires from instrumental, fear-based motivations. Anticipatory intrinsic satisfaction directly measures the intrinsic affective anticipation characteristic of autonomous motivation. Value alignment assesses integrated regulation, the highest form of autonomous motivation where caregiving becomes part of one’s identity.

##### 3.4.3.2 Inner needs

Aligned with SDT’s three basic psychological needs, this construct measures how pet ownership may satisfy: (a) autonomy—the volitional choice in pet selection, care routines, and lifestyle adjustments without external coercion; (b) competence—the mastery experiences in feeding, health management, and problem-solving that build efficacy beliefs; and (c) relatedness—the emotional bonds and unconditional responses that fulfill belongingness needs. The fulfillment of these needs is hypothesized to activate intrinsic motivation toward parenthood by demonstrating that caregiving can be a self-determined, efficacious, and relationally rewarding experience.

##### 3.4.3.3 Emotional value

This construct captures the affective benefits derived from pet ownership that are distinct from motivational processes. While motivation concerns the “why” of behavior, emotional value concerns the “how it feels.” Pet ownership provides immediate positive emotions (warmth, happiness, security) and builds confidence in family life capabilities. These emotional resources reduce the perceived burden and anxiety associated with childbearing, thereby indirectly supporting fertility intentions without directly measuring them. This distinction preserves the theoretical separation between emotional experience and behavioral intention.

#### 3.4.4 Control variables

Drawing on existing research identifying key factors associated with fertility intentions, demographic characteristics (gender, age, education level, occupation) and life circumstances/economic characteristics (academic/work demands, living conditions, monthly income, household registration and residence type) were included to eliminate interference from unrelated variables on the core relationship between pet ownership behavior and fertility intentions(as shown in Table 1).

**Table 1:** Variable selection.

Variable type	Latent variable	Observed variables	Questionnaire item
Outcome variable	Fertility intention	Fertility planning	I plan to have children in the future
		Perception of parenting responsibilities	I am willing to take on the responsibility of raising a child.
		Emotional expectations for parenthood	I look forward to having my own children.
		Understanding of family integrity	I believe having two children contributes to greater family wholeness.
		Children’s interaction needs	I look forward to the companionship and shared growth between siblings.
Explanatory variables	Pet ownership behavior	Pet ownership status (whether pets are owned)	Do you currently own a pet?
		Number of pets owned	How many pets do you own?
		Pet ownership atmosphere in your social circle	Do your relatives and friends keep pets?
		Preferred pet type	Your preferred pet type is (mammals/birds/reptiles/aquatic animals)
		Time and financial commitment to pet ownership	I am willing to invest time and money in my pet
		Pet companionship activities	I proactively spend time with my pet daily.
		Importance of pet ownership	I consider pet ownership an important part of my life.
Mediating variables	Motivation for childbearing	Identified regulation	Caring for my pet has deepened my appreciation for nurturing life, strengthening my desire to experience parenthood.
		Integrated regulation	I look forward to building a meaningful parent–child relationship, not merely securing practical support.
		Intrinsic motivation	Imagining myself as a parent brings me genuine fulfillment and joy.
		Value alignment	Raising my pet has shown me that nurturing aligns with my core values.
	Inner needs	Competence	Caring for my pet has built my confidence in nurturing life.
		Autonomy	I choose how to care for my pet, which strengthens my sense of control over family life.
		Relatedness	My pet makes me feel connected and needed, laying groundwork for desiring parent–child bonds.
		Competence	Managing my pet’s needs convinces me I can handle parenthood.
	Emotional value	Positive emotional experiences through pet ownership	Interacting with pets makes me feel warm and happy
		Parental emotional transfer (anticipation of parent–child interaction)	I look forward to doing similar activities with my future child (e.g., playing, spending time together).
		Parental emotional transference (aspirations for shared growth)	I long for the warm moments of growing together with my child
		Enhanced confidence in family life	Pets give me greater confidence in my future family life.
		Recognition of the connection between emotional value from pet ownership and family happiness	The emotional value derived from pet ownership can enhance family happiness.
Controlling variables	Demographic characteristics: Gender, Age, Education Level, Occupation Life circumstances and economic characteristics: academic/work demands, living conditions, monthly income, household registration/residence type		

### 3.5 Reliability and validity tests

SPSS 24.0 software was used to perform descriptive statistics, reliability analysis (Cronbach’s α), Pearson correlation analysis, and control variable processing. AMOS 24.0 software was employed for structural equation modeling to examine the measurement model and hypothesized pathways.

This study employed Cronbach’s α to assess internal consistency of the scales, with results presented in Table 2. The Cronbach’s α coefficients for each latent variable fell within the ideal range of 0.7–1.0: pet ownership behavior (0.816), reproductive motivation (0.826), intrinsic needs (0.855), emotional value (0.856), and reproductive intention (0.747). This indicates that the scales employed exhibit good internal consistency, high reliability, and stable, dependable measurement outcomes.

**Table 2:** Reliability and validity analysis.

Indicators	Pet ownership behavior	Motivation for childbearing	Inner needs	Emotional value	Fertility intention
Cronbach’s α	0.816	0.826	0.855	0.856	0.747

As shown in the analysis results of Table 3, in this validity test of the fertility intention scale, the AVE values for all dimensions exceeded 0.5, and the CR values also surpassed 0.7. This indicates that each dimension possesses good convergent validity and composite reliability.

**Table 3:** Convergent validity analysis.

Path relationships	Estimate	AVE	CR
YC1 ← Pet ownership behavior	0.718	0.527	0.817
YC2 ← Pet ownership behavior	0.711		
YC3 ← Pet ownership behavior	0.759		
YC4 ← Pet ownership behavior	0.716		
DJ1 ← Motivation for childbearing	0.76	0.543	0.826
DJ2 ← Motivation for childbearing	0.733		
DJ3 ← Motivation for childbearing	0.761		
DJ4 ← Motivation for CHILDBEARING	0.692		
XY1 ← Inner needs	0.75	0.597	0.856
XY2 ← Inner needs	0.781		
XY3 ← Inner needs	0.818		
XY4 ← Inner needs	0.74		
QX1 ← Emotional value	0.751	0.544	0.856
QX2 ← Emotional value	0.724		
QX3 ← Emotional value	0.758		
QX4 ← Emotional value	0.738		
QX5 ← Emotional value	0.716		
SY1 ← Fertility intentions	0.672	0.5	0.75
SY2 ← Fertility intention	0.754		
SY3 ← Fertility intention	0.694		

## 4 Results

### 4.1 Descriptive characteristics

#### 4.1.1 Basic information about the respondent

As shown in Table 4, the sample demonstrates a relatively balanced gender distribution, with females accounting for 51.9% and males for 48.1%. In terms of educational attainment, the majority of respondents held university degrees (36.5%), followed by secondary school (22.3%) and postgraduate qualifications (15.1%), while only a small proportion (4.7%) had lower secondary education. This educational profile indicates that the sample is predominantly composed of individuals with moderate to high educational backgrounds.

**Table 4:** Basic characteristics of the sample.

Dimension	Percentage%
Gender	
Male	48.1
Female	51.9
Education	
Lower secondary school	4.7
Secondary school	22.3
University	36.5
Postgraduate	15.1
Busy with studies/work	
Yes	86.8
No	13.2
Age	
18–27	49.5
28–35	19.6
36–45	18.3
>46	12.6
Marital and relationship status	
Single	24.7
Married with no children	20.5
Married with children	53.2
Romance	1.6
Monthly income	
<3,000	41.2
3,000–5,000	16.7
5,000–8,000	29.8
8,000以上	12.3

Regarding occupational status, a substantial majority (86.8%) reported being busy with studies or work, reflecting the typical lifestyle characteristics of young and middle-aged adults in contemporary China. With respect to marital and relationship status, more than half of the respondents (53.2%) were married with children, while 20.5% were married without children, and 24.7% were single. Notably, only 1.6% of respondents were in romantic relationships but unmarried, suggesting that marriage and childbearing remain closely linked in the Chinese context.

In terms of economic characteristics, the monthly income distribution reveals that 41.2% of respondents earned less than 3,000 yuan, while 29.8% earned between 5,000 and 8,000 yuan. Only 12.3% reported monthly incomes exceeding 8,000 yuan, indicating that the sample predominantly represents middle- to lower-income groups. Additionally, Age was measured as an ordinal variable with four categories: 1 = 18–27 years, 2 = 28–35 years, 3 = 36–45 years, and 4 = over 46 years. The mean value of 3.56 (SD = 1.534) indicates that the average respondent falls between the 28–35 and 36–45 age brackets. The sample includes 69.1% of respondents of reproductive age (18–35 years) and 30.9% aged 36 years and above.

While our sample includes a non-negligible proportion of middle-aged and elderly participants (30.9%), which reflects the natural demography of pet owners, we acknowledge that the psychological pathways linking pets to fertility intentions may differ across age groups. Encouragingly, our sub-group analysis confirmed the main hypotheses hold in the reproductive-age subsample. However, the presence of older participants offers a unique opportunity for future research to explore the moderating role of age—specifically, whether pet ownership serves as a ‘substitute for children’ in older adults, versus a ‘preparation for children’ in younger adults. We have added this as a key direction for future inquiry.

Overall, the demographic composition of the sample aligns with the study’s objective of investigating fertility intentions among young and middle-aged adults. The diversity in educational backgrounds, income levels, and marital statuses ensures sufficient variability for examining the relationship between pet ownership behavior and fertility intentions while controlling for potential confounding factors.

#### 4.1.2 Pet ownership overview

As Table 5 indicates, based on 551 valid survey responses, the pet ownership rate among respondents reached 61.2%, with over 60% having pet-owning experience. This figure significantly exceeds traditional perceptions, indicating that pet ownership has evolved from an “optional lifestyle choice” to a “mainstream component of life,” with pets playing an increasingly vital role within families. From a demographic perspective, gender differences in pet ownership remain pronounced: female pet owners account for 55.5%, compared to 44.5% for males. Age-wise, the 20–45 age group forms the core of pet ownership, reflecting a strong correlation between pet ownership and economic stability—young and middle-aged adults typically enjoy more stable incomes and lifestyles, making them better equipped to shoulder the responsibilities and costs of pet care.

**Table 5:** Sample pet ownership characteristics.

Variable	Number of respondents	Percentage
Pet ownership		
Yes	337	61.2%
No	214	38.8%
Number of pets owned		
1	164	29.8%
2	81	14.7%
3	51	9.3%
4 or more	41	7.4%
Do relatives or friends keep pets?		
Yes	167	30.3%
No	384	69.7%

Regarding pet numbers and types, a distinct preference for fewer, higher-quality companions emerged. Pet owners average 1.91 pets per household, with 48.7% owning one pet, 24% owning two, and 27.3% owning three or more. This distribution aligns with urban space constraints while reflecting owners’ pursuit of meticulous care, avoiding diminished quality due to excessive numbers. Preferences for pet types show significant variation. Traditional mammalian pets (cats, dogs, hamsters, etc.) hold absolute dominance, favored by 83.6% of respondents. This preference is closely tied to the high interactivity and strong emotional feedback these pets provide. Aquatic pets (fish, turtles, etc.), reptiles (snakes, lizards, etc.), and birds (parrots, etc.) accounted for 21.4, 25.4, and 29% of preferences, respectively. While niche pet types hold some market share, they primarily serve as supplementary choices requiring “low interaction demands”.

Regarding attitudes toward pet ownership and willingness to invest in pets, respondents generally placed high importance on their companions. The highest average score (3.99) was for the statement “Pet ownership is an important part of life.” Nearly 80% of respondents explicitly recognized pets’ value in daily life, viewing them as “important family members.” The statements “I actively spend time with my pet daily” (3.82) and “I am willing to invest time and money in my pet” (3.89) followed closely. The statement “I don’t feel pet ownership significantly impacts my life” scored 3.67 points, indicating most people effectively balance pet care with work, socializing, and daily routines without experiencing significant burdens. Additionally, 69.7% of respondents indicated that their relatives and friends do not own pets, while only 30.3% have a social circle of pet owners.

#### 4.1.3 Fertility overview

The current fertility landscape is characterized by a predominance of first-time births and a relatively low second-child rate. Among the 551 respondents, 55.9% had given birth to one child, while 44.1% had not yet had their first child. Age distribution reveals a pronounced concentration of first births: the highest first-child birth rate (72.2%) occurs among those aged 36–46 and older, followed by the 28–35 age group (68.5%). In contrast, the birth rate among those aged 23–28 was only 50%.

Regarding second-child births, the overall fertility rate stands at just 30%. Among respondents who have already had one child, only 39.6% chose to have a second child. Furthermore, 62.2% of families with one child do not agree that having two children contributes to family completeness. The overall fertility intention remains at a moderately high level. Based on a comprehensive score assessment across three core dimensions—“plans to have children,” “willingness to assume parenting responsibilities,” and “desire to have children”—the average score was 3.91 out of 5. A total of 89.1% of respondents scored between 3 and 5 points, while only 10.9% scored below 3 points (as shown in Table 6).

**Table 6:** Sample fertility characteristics.

Fertility intention indicators	Number of respondents (persons)	Percentage (%)	Mean	Standard deviation S. E.
Planned childbearing				
1–Strongly disagree	22	4%	3.93	1.047
2-Somewhat disagree	34	6.2%		
3-Not necessarily	87	15.8%		
4-Somewhat agree	224	40.7%		
5-Strongly agree	184	33.4%		
Awareness of parenting responsibilities				
1-Strongly disagree	12	2.2%	4.1	0.989
2-Somewhat disagree	26	4.7%		
3-Not necessarily	94	17.1%		
4-Somewhat agree	182	33.0%		
5-Strongly agree	237	43.0%		
Emotional expectations regarding childbirth				
1-Strongly disagree	20	3.6%	3.69	1.112
2-Somewhat disagree	74	13.4%		
3-Not necessarily	111	20.1%		
4-Somewhat agree	199	36.1%		
5-Strongly agree	147	23.7%		

#### 4.1.4 Analysis of the relationship between pet ownership and childbearing

Table 7 shows the Relationship Between Childbearing and Pet Ownership. Economic pressure is a key constraint in childbearing decisions. Combined with image data, 81.7% of respondents believe that “the financial burden of raising children is significantly greater than that of keeping pets,” while only 18.3% hold the opposite view (that pet ownership is more costly).

**Table 7:** Analysis of the relationship between childbearing and pet ownership.

Dimension	Number of respondents	Percentage (%)
Financial pressure		
Greater pressure from pet ownership	101	18.3%
Raising children is more stressful	450	81.7%
If having a child means giving up your pet		
Would give up pets without hesitation	148	26.9%
Would be torn, but might ultimately give up the pet	228	41.4%
Trying to find a balance, do not want to give up	122	22.1%
Refuse to give up pet ownership, opting to adjust family planning instead	53	9.6%

When presented with the hypothetical trade-off of “giving up a pet due to childbirth”: 26.9% of respondents stated they would “give up the pet without hesitation,” 41.4% were “very conflicted but might ultimately give up the pet,” 22.1% would “try to find a balance and not give up the pet,” while 9.6% were “firmly unwilling to give up the pet and would instead alter their childbirth plans.” The data distribution reveals that while 68.3% of respondents (26.9% + 41.4%) might lean toward giving up their pet in such a trade-off, 31.7% (22.1% + 9.6%) would adjust their childbearing plans to retain their pet.

### 4.2 Empirical analysis results

#### 4.2.1 Reliability and validity tests

As shown in Table 8, the standardized correlation coefficients between the two dimensions are both less than the square root of the AVE value for that dimension, indicating that each dimension possesses good discriminant validity.

**Table 8:** Distinguishing validity analyses.

Indicators	Pet ownership behavior	Motivation for childbearing	Inner needs	Emotional value	Fertility intention
Pet ownership behavior	0.572				
Reproductive drive	0.351	0.543			
Inner needs	0.23	0.328	0.597		
Emotional value	0.262	0.358	0.352	0.544	
Fertility intention	0.512	0.448	0.294	0.356	0.5
Square root of AVE	0.756	0.737	0.773	0.738	0.707

The results of Pearson correlation analysis among dimensions (as shown in Table 9) indicate that all correlation coefficients between dimensions are statistically significant at the 0.01 level (two-tailed). Among these, the correlation coefficient between pet ownership behavior and fertility intention is the highest (0.406), followed by that between fertility motivation and fertility intention (0.349). The correlation coefficient between intrinsic needs and pet ownership behavior is relatively low (0.196) but remains statistically significant. The significant correlations among dimensions provide a foundation for subsequent path analysis.

**Table 9:** Pearson correlation analysis.

Dimension	Average pet ownership score	Average motivation score	Average need score	Average emotional score	Average fertility score
Average pet ownership score	1				
Drive average score	0.290**	1			
Average need score	0.196**	0.280**	1		
Emotional average score	0.221**	0.296**	0.300**	1	
Fertility average score	0.406**	0.349**	0.231**	0.279**	1

**Significant at the 0.01 level (two-tailed).

Based on the theoretical framework and characteristics of observed variables, a structural equation model was constructed to examine how pet ownership influences fertility intentions. Model fit results are presented in Table 10. All fit indices meet ideal standards: Root Mean Square Error of Approximation (RMSEA) was 0.033, below the excellent standard of 0.05, indicating minimal approximation error; Goodness-of-Fit Index (GFI) was 0.955, and Adjusted Goodness-of-Fit Index (AGFI) was 0.943, both exceeding the excellent threshold of 0.9, reflecting high overall model fit; Among the incremental fit indices, the IFI was 0.977, NFI was 0.941, and CFI was 0.977, all meeting the excellent requirement of >0.9, indicating excellent fit of the model’s internal structure. In summary, the model exhibits good overall fit and is suitable for subsequent path analysis (see Figure 1 for full model).

**Table 10:** Model fit test.

Indicator	Reference standard	Observed value	Fit evaluation
CMIN/DF	1–3: Excellent, 3–5: Good	1.602	Ideal
RMSEA	<0.05 is excellent, <0.08 is good	0.033	Ideal
GFI	>0.9 is Excellent, >0.8 is Good	0.955	Ideal
AGFI	>0.9 is excellent, >0.9 is good	0.943	Ideal
IFI	>0.9 is excellent, >0.10 is good	0.977	Ideal
NFI	>0.9 is excellent, >0.11 is good	0.941	Ideal
CFI	>0.9 is excellent, >0.12 is good	0.977	Ideal

**Figure 1:**
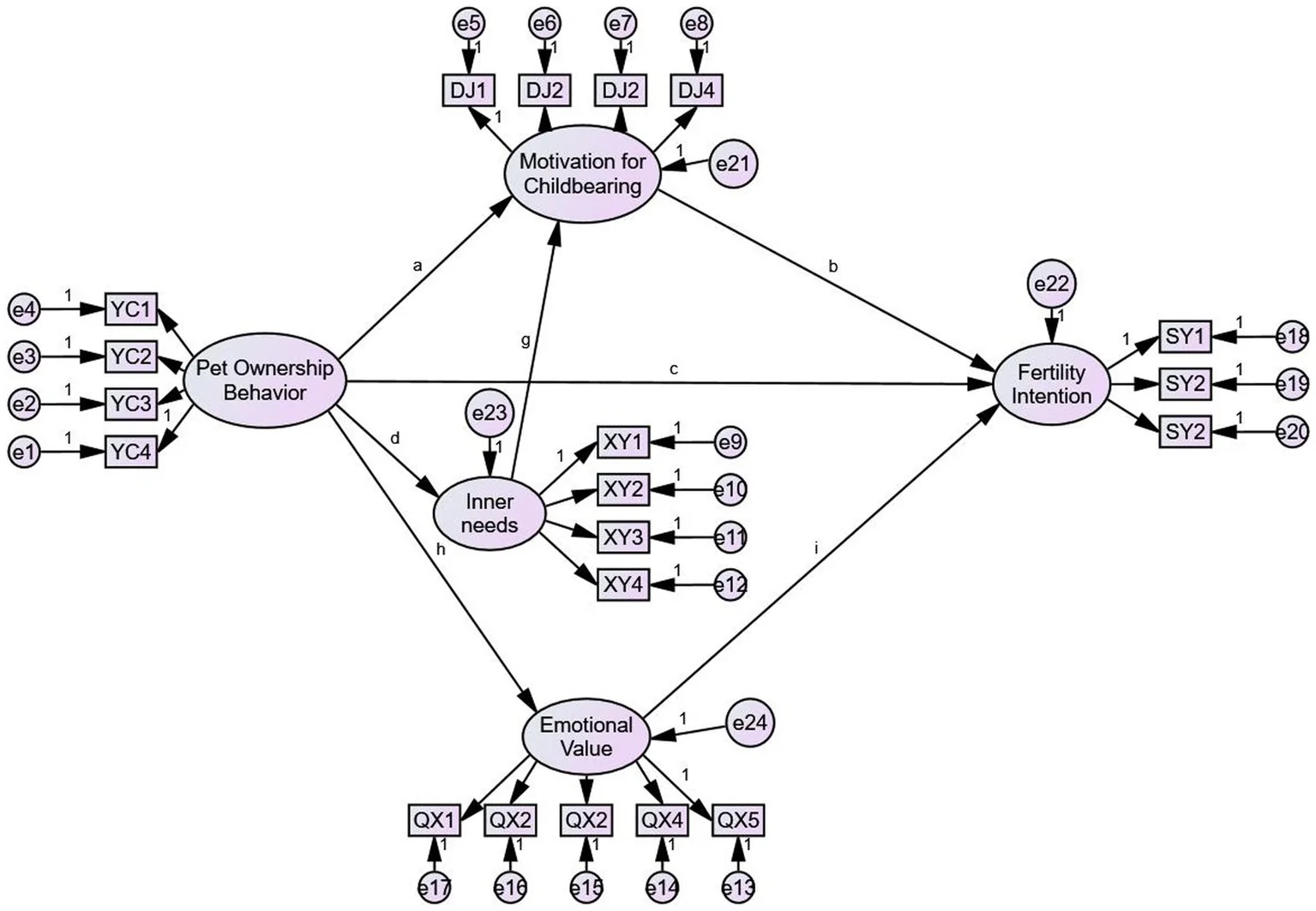
Structural equation modelling diagram.

### 4.3 Path analysis of pet ownership behavior on fertility intentions

Empirical results indicate that the direct coefficient of pet ownership on fertility intentions is 0.383 (*p* < 0.001), representing the strongest association among all pathways. This demonstrates that pet ownership directly shapes individual fertility intentions without relying on mediating variables.

The positive coefficient of pet ownership on fertility motivation is 0.302 (*p* < 0.001), and the positive coefficient of fertility motivation on fertility willingness is 0.262 (*p* < 0.001). The indirect association of this path is 0.302 × 0.262 ≈ 0.091 (95% confidence interval [0.048, 0.152], *p* = 0.000), representing the strongest single mediating pathway among all pathways.

The positive coefficient of pet ownership behavior on intrinsic needs is 0.254 (*p* < 0.001), the positive coefficient of intrinsic needs on reproductive motivation is 0.256 (*p* < 0.001), and the positive coefficient of reproductive motivation on reproductive willingness is 0.262 (*p* < 0.001). The chained indirect association of this pathway was 0.254 × 0.256 × 0.262 ≈ 0.017 (corresponding to ind2 = 0.015 in the mediation effect table; the discrepancy stems from rounding of decimal places), with a 95% confidence interval [0.006, 0.035] and *p* = 0.000. This indicates that intrinsic needs and reproductive motivation form a chained mediation, jointly transmitting the influence of pet ownership behavior on reproductive intention.

The positive coefficient of pet ownership on emotional value is 0.291 (*p* < 0.001), while the positive coefficient of emotional value on fertility intention is 0.164 (*p* = 0.001), with an indirect association of 0.291 × 0.164 ≈ 0.048 (corresponding to indirect effect 2 = 0.042 in the mediation table, the discrepancy stems from rounding of decimal places), with a 95% confidence interval [0.016, 0.085] and *p* = 0.001. This indicates that emotional value plays a significant mediating role between pet ownership and fertility intentions (as shown in Tables 11, 12).

**Table 11:** Structural equation path relationship results.

Path Relationships	Estimate	S. E.	C. R.	P	Estimate(age)
Inner needs ← Pet ownership	0.254	0.056	4.908	***	0.356
Motivation for childbearing ← Pet ownership	0.302	0.054	5.687	***	0.240
Emotional value ← Pet ownership	0.291	0.052	5.545	***	0.292
Motivation for childbearing ← Inner need	0.256	0.047	5.017	***	0.195
Fertility intention ← Motivation for childbearing	0.262	0.048	4.772	***	0.269
Fertility intention ← Emotional value	0.164	0.045	3.242	0.001	0.162
Fertility intention ← Pet ownership	0.383	0.054	6.261	***	0.256

***Indicates the significant at the 1% level.

**Table 12:** Mediation effects results.

Parameter	Path	Estimate	Lower	Upper	P
Indirect effect 1	a*b	0.070	0.035	0.126	0.000
Direct effect 1	c	0.338	0.223	0.461	0.001
Total effect 1	a*b + c	0.408	0.288	0.536	0.001
Indirect effect 2	h*i	0.042	0.016	0.085	0.001
Direct effect 2	c	0.338	0.223	0.461	0.001
Total effect 2	h*i + c	0.380	0.270	0.508	0.001
ind1	a*b	0.070	0.035	0.126	0.000
ind2	d*g*b	0.015	0.006	0.035	0.000
totind	Ind1 + ind2	0.085	0.042	0.149	0.001
total	c + totind	0.423	0.300	0.553	0.001

To account for potential demographic confounding, age was incorporated as a control variable into the structural equation model. As presented in the rightmost column of Table 11, the inclusion of age substantially altered the magnitude of several path coefficients while preserving their directional consistency and statistical significance. Most notably, the direct effect of pet ownership on fertility intentions decreased from 0.383 to 0.256 (*p* < 0.001), suggesting that a non-trivial portion of the direct association observed in the baseline model was attributable to age-related demographic factors.

Conversely, the effect of pet ownership on intrinsic needs increased from 0.254 to 0.356 (*p* < 0.001) after controlling for age, indicating that the satisfaction of autonomy, competence, and relatedness through pet care is particularly pronounced among younger cohorts, and that controlling for age unmasks this stronger underlying psychological mechanism. The effect on fertility motivation attenuated from 0.302 to 0.240 (*p* < 0.001), while the paths from emotional value to fertility intentions (0.162, *p* = 0.001) and from fertility motivation to fertility intentions (0.269, *p* < 0.001) remained virtually unchanged. The stability of these mediating pathways underscores the robustness of the proposed psychological transmission mechanisms independent of age-related demographic variation.

### 4.4 Robustness test

To validate the reliability and stability of the structural equation model results presented earlier, this study employed the “sample structure adjustment method” for robustness testing. The original sample contained a relatively high proportion of individuals with bachelor’s degrees or higher, which could potentially introduce bias in the research conclusions due to the sample distribution characteristics of the education dimension. First, samples with bachelor’s degrees or higher were selected; then, 50% of these were randomly removed, yielding a subsample of 284 cases. The structural equation model was rebuilt on this subsample and subjected to regression analysis to test the stability of core path relationships.

Regarding core path coefficients (see Table 13), the direct effect coefficient of pet ownership on fertility intention was 0.423 (*p* < 0.001). Although slightly fluctuating compared to the original model’s 0.383, it maintained a significant positive relationship. In the mediating pathways, the coefficients for pet ownership’s association with on reproductive motivation, intrinsic needs, and emotional value were 0.327 (*p* < 0.001), 0.210 (*p* = 0.003), and 0.226 (*p* = 0.002), respectively. The transmission coefficients from reproductive motivation and emotional value to reproductive willingness were 0.245 (*p* = 0.001) and 0.210 (*p* = 0.003), respectively. The chain transmission coefficient from intrinsic needs → reproductive motivation was 0.264 (*p* < 0.001). The signs and significance levels of these coefficients remained consistent with the original model, with numerical fluctuations controlled within 15% of the original values, indicating no substantive differences.

**Table 13:** Robust structural equation results.

Path relationships	Estimate	S. E.	C. R.	*p*	Label
Inner needs ← Pet ownership	0.21	0.067	2.955	0.003	d
Motivation for childbearing ← Pet ownership	0.327	0.074	4.704	***	a
Emotional value ← Pet ownership	0.226	0.065	3.163	0.002	h
Motivation for childbearing ← Inner need	0.264	0.077	3.898	***	g
Fertility intention ← Motivation for childbearing	0.245	0.051	3.256	0.001	b
Fertility intention ← Emotional value	0.21	0.056	2.963	0.003	i
Fertility intention ← Pet ownership	0.423	0.062	4.902	***	c

***Indicates the significant at the 1% level.

Further comparing the magnitude of mediating effects in Table 14, the single mediating effect of reproductive motivation in the subsample was 0.058 (95% confidence interval [0.017, 0.132]), while that of emotional value was 0.034 (95% confidence interval [0.008, 0.081]). The chained mediating effect from intrinsic needs to reproductive motivation was 0.010 (95% confidence interval [0.002, 0.038]), and the total mediating effect was 0.068 (95% confidence interval [0.020, 0.149]). Although the specific values of the mediating effects differed slightly from the original model, the effect directions were consistent, and all passed the significance test. This indicates that the mediating mechanism through which pet ownership is associated with fertility intentions did not change due to the adjustment of the sample’s educational structure.

**Table 14:** Robust mediation effect results.

Parameter		Estimate	Lower	Upper	*p*
Indirect Effect 1	a*b	0.058	0.017	0.132	0.008
Direct effect 1	c	0.304	0.176	0.446	0.001
Total Effect 1	a*b + c	0.362	0.229	0.505	0.001
Indirect effect 2	h*i	0.034	0.008	0.081	0.009
Direct effect 2	c	0.304	0.176	0.446	0.001
Total Effect 2	h*i + c	0.338	0.208	0.476	0.001
ind1	a*b	0.058	0.017	0.132	0.008
ind2	d*g*b	0.01	0.002	0.038	0.007
totind	Ind1 + ind2	0.068	0.02	0.149	0.008
total	c + totind	0.372	0.237	0.521	0.001

### 4.5 Heterogeneity test

To examine whether there are group differences in the impact of pet ownership on fertility intentions, this study divided the sample into a pet-owning group (*N* = 337) and a non-pet-owning group (*N* = 214), conducting separate OLS regression analyses. The results are presented in the Table 15.

**Table 15:** Heterogeneity effect results.

Variable	pet groupβ	pet group p	No-pet group β	No-pet group p
Pet ownership behavior	0.239	0.000***	0.398	0.000***
Motivation for childbearing	0.243	0.000***	0.173	0.039**
Inner needs	0.084	0.062*	0.042	0.538
Emotional value	0.112	0.052*	0.146	0.027**

*Indicates the significant at the 10% level. **Indicates the significant at the 5% level. ***Indicates the significant at the 1% level.

From the perspective of core explanatory variables, pet ownership behavior exhibits a significant positive impact on fertility intentions in both groups; however, the coefficient in the non-pet ownership group (β = 0.398, *p* < 0.001) is markedly higher than that in the pet ownership group (β = 0.239, *p* < 0.001). These findings indicate that for individuals who have not yet owned pets, their pet ownership tendency or attitude plays a more prominent predictive role in fertility intentions, suggesting that pet ownership attitude may serve as a precursor signal triggering fertility intention.

From the perspective of mediation mechanisms, the coefficient for fertility motivation was significantly higher in the pet-keeping group (β = 0.243, *p* < 0.001) than in the non-pet-keeping group (β = 0.173, *p* < 0.05), indicating that the pathway through which pet ownership activates fertility motivation and subsequently enhances fertility intention is more pronounced among pet owners, thereby confirming the existence of the “pet ownership → fertility motivation → fertility intention” transmission mechanism. Emotional value exhibited significant positive effects in both groups, serving as a universal mechanism across populations. Intrinsic needs did not reach conventional significance levels in either group, suggesting they primarily exert their influence indirectly via fertility motivation, consistent with the chain-mediation hypothesis.

To further examine whether there are age-related differences in the impact of pet ownership on fertility intentions, this study divided the sample into four age groups: 18–27 years (*N* = 273), 28–35 years (*N* = 108), 36–45 years (*N* = 101) and 46 years and over (*N* = 69), and conducted OLS regression analyses for each group; the results are shown in Table 16.

**Table 16:** Age heterogeneity effect results.

Variable	age18-27	age28-35	age36-45	age>46
Pet ownership behavior	0.245***	0.420***	0.312***	0.399**
Motivation for childbearing	0.288***	0.162	−0.023	0.251*
Inner needs	−0.056	0.100*	0.333**	0.116
Emotional value	0.122**	0.226**	0.093	0.091

*Indicates the significant at the 10% level. **Indicates the significant at the 5% level. ***Indicates the significant at the 1% level.

With regard to the core explanatory variables, pet ownership had a significant positive effect on fertility intentions across all four groups, although the strength of the effect exhibited an ‘inverted U-shaped’: the effect was strongest among the 28–35 age group (β = 0.420, *p* < 0.001), followed by those aged 46 and over (β = 0.399, *p* < 0.05) and the 36–45 age group (β = 0.312, *p* < 0.001), with the 18–27 age group showing the relatively weakest effect (β = 0.245, *p* < 0.001). This suggests that, as the 28–35 age group represents a critical window for fertility decisions, the ‘rehearsal for parenthood’ effect of pet ownership is most pronounced during this period, with individuals being more likely to transfer their pet-ownership experience to their family planning.

In terms of mediating mechanisms, significant differentiation in the mechanisms at work was observed across different age groups. Fertility motivation was significant only in the 18–27 age group (β = 0.288, *p* < 0.001), suggesting that young people are more likely to use pet ownership to activate the idealized motivation of ‘I want to become a parent’; emotional value was significant in the 18–35 age group (β = 0.122, *p* < 0.05; β = 0.226, *p* < 0.05), suggesting that young people are more likely to translate the emotional fulfillment derived from pet ownership into a desire to have children; whereas intrinsic needs were significant only in the 36–45 age group (β = 0.333, *p* < 0.01), indicating that middle-aged individuals rely more on pet ownership to satisfy their needs for autonomy, competence and relatedness in order to drive their decisions regarding childbearing. Among those aged 36–45, the effect of fertility motivation was neither significant nor positive (β = −0.023, *p* = 0.834), which may stem from the fact that individuals in this age group have already finalized their fertility decisions, thereby reducing the marginal activating effect of pet ownership on fertility motivation.

## 5 Discussion

### 5.1 Direct path: pet ownership → fertility intentions

The empirical results indicate a direct positive association between pet ownership and fertility intentions, representing the strongest effect among all pathways. The daily care (e.g., feeding, health management), emotional companionship, and responsibility-bearing involved in pet ownership essentially constitute an early adaptation to the “family caregiving role.” Pets are assigned a “child-like” role (Marks et al., 1994), making pet ownership a crucial practice ground for young adults to rehearse “parental responsibilities.” Individuals gradually develop awareness and acceptance of “long-term commitment” and “emotional investment,” which is associated with reducing anxiety about post-childbirth role transitions. This association is consistent with previous research suggesting that caregiving experiences with pets may enhance family values and acceptance of parenting roles. This directly relate to fertility intentions (Volsche and Gray, 2016), thus validating Hypothesis H1.

### 5.2 Pet ownership → motivation for childbearing → fertility intention

The results suggest that pet ownership is associated with fertility motivation, which in turn relates to fertility willingness. Pet ownership provides a prototypical autonomy-supportive context: individuals voluntarily choose to engage in caregiving without external coercion, make self-directed decisions regarding pet care methods and schedules, and experience their actions as originating from personal choice rather than social obligation (Hope et al., 2014). According to SDT, such autonomy-supportive contexts facilitate the transformation of initially enjoyable activities into identified and integrated regulation, forms of autonomous motivation where behavior becomes linked to personal values and self-identity (Ryan and Deci, 2017).

Specifically, pet ownership cultivates autonomous fertility motivation through the satisfaction of three basic psychological needs (Junça-Silva, 2022). The first need is autonomy, manifested in pet ownership through volitional choice in pet selection, care routines, and lifestyle adjustments, all in the absence of external pressure or social surveillance. This satisfaction fosters the belief that parenthood can similarly be a freely chosen life path, characterized by the sentiment that one chooses to nurture. The second need is competence, manifested through mastery experiences in feeding, health management, behavior training, and problem-solving, with observable outcomes of successful care. This builds transferable efficacy beliefs, the conviction that one can care for dependents, reducing anxiety about parenting capability and enhancing anticipatory confidence in the parental role. The third need is relatedness, manifested through receipt of unconditional, non-judgmental emotional responses from pets and the formation of secure attachment bonds. This satisfies belongingness needs while creating a psychological foundation for desiring deeper, reciprocal human attachment. The emotional cycle of being needed, responding, and being responded to becomes a valued relational pattern that individuals wish to replicate with children (He and Liang, 2024).

When these three needs are concurrently satisfied through pet ownership, individuals undergo a motivational shift: childbearing is no longer perceived as a social norm to comply with, which would represent controlled motivation, but becomes a self-concordant goal, representing autonomous motivation, that aligns with their emerging identity as nurturing, competent, and relationally fulfilled persons (Guo et al., 2021). This transformation explains why the path through autonomous fertility motivation represents the strongest indirect effect in our model: it captures not merely the acquisition of caregiving skills or the reduction of anxiety, but the fundamental reorganization of reproductive desires around personally endorsed values. Thus, Hypothesis H2 holds.

### 5.3 Pet ownership → inner needs → motivation for childbearing → fertility intention

The chained mediation through intrinsic needs and fertility motivation suggests that pet ownership may relate to fertility intentions by fulfilling fundamental psychological needs. Regarding the fulfillment of intrinsic needs, pet ownership fills emotional connection gaps through stable companionship and interaction, satisfying the core need for “relatedness” (Kanat-Maymon et al., 2016). Simultaneously, autonomous decision-making during pet care—such as choosing care methods and adjusting life plans—further fulfills the intrinsic desire for “autonomy” in life management. The dog-owner bond exhibits attachment characteristics similar to human infant-caregiver relationships, providing a secure base that supports autonomous exploration (Prato-Previde et al., 2003). Once these core intrinsic needs are satisfied, individuals’ yearning for family life intensifies, thereby activating reproductive motivation. This transforms childbearing from a social norm into a proactive choice driven by self-fulfillment, ultimately boosting fertility intentions through the transmission of reproductive motivation. Hypothesis H3 holds.

### 5.4 Pet ownership → emotional value → fertility intention

Pets provide immediate emotional comfort through daily interactions. For instance, during periods of high stress, a pet’s companionship can alleviate anxiety and soothe negative emotions (McConnell et al., 2011), establishing a stable emotional support mechanism. This positive emotional state reduces the negative impact of childbearing on individuals’ perceived life pressures, diminishing the aversion to childbearing stemming from emotional burdens. Simultaneously, the long-term accumulation of positive emotions enhances individuals’ life satisfaction and happiness, bolstering their confidence to tackle the various challenges of childbearing. This, in turn, indirectly promotes an increase in fertility intentions, confirming Hypothesis H4.

### 5.5 Study limitations and future directions

Several limitations of this study should be acknowledged. First, the cross-sectional design precludes causal inferences; reverse causality is equally plausible, as individuals with stronger family orientation or higher fertility intentions may be more likely to own pets. Second, the sample was recruited through an online survey platform and social media, which may introduce self-selection bias toward individuals with higher education levels, urban residence, and stronger internet engagement. The predominance of female (55.5%) and unmarried (54.1%) respondents further limits generalizability. Third, while we have revised the measurement items to reduce conceptual overlap, some degree of shared method variance may still exist given the self-report nature of all variables. Fourth, the adapted items for non-pet owners assess hypothetical rather than actual behaviors, which may introduce measurement error. Also, our measurement approach relied on 5-point Likert scales with predominantly unidirectional items, which may be vulnerable to acquiescence response bias. Although our post-hoc analyses (Harman‘s single-factor test and latent method factor control in SEM) indicated that common method variance did not substantially affect our core findings, we cannot completely rule out the possibility of response style effects. Future research could employ balanced scales with both positively and negatively worded items, or alternative response formats such as semantic differential scales, to further mitigate acquiescence bias. Additionally, collecting data from multiple sources (e.g., partner reports) could help reduce concerns about same-source bias. Future research should employ longitudinal designs, probability sampling methods, and objective measures to address these limitations.

## 6 Conclusion

Based on self-determination theory, this study systematically analyzed theoretical frameworks and conducted empirical research to examine the association between pet ownership and young adults’ fertility intentions and their underlying mechanisms. Findings reveal that pet ownership not only directly associated with fertility intentions but also indirectly linked to them through mediating variables such as reproductive motivation, intrinsic needs, and emotional value, thereby illuminating the complex relationship between pet ownership and fertility intentions. However, it is equally plausible that individuals with stronger pre-existing family orientation self-select into pet ownership, or that pets serve as partial substitutes for children when structural barriers to childbearing are high. Specifically: Pet ownership provides opportunities for daily caregiving, emotional companionship, and responsibility-taking, which is associated with more positive expectations about childbearing. Through routine care and emotional exchange during pet ownership, individuals pre-adapt to caregiving responsibilities within a family context. This reduces anxieties about future role transitions associated with childbirth, thereby relating to their willingness to have children. Yet for individuals facing severe economic or housing constraints, the same caregiving experiences may reinforce the perception that nurturing needs are already met, potentially reducing rather than enhancing fertility intentions. These findings suggests that pets and children may function as competing commitments for a substantial minority. Secondly, the parenting experience and emotional bonds cultivated through pet ownership activate individuals’ intrinsic motivation toward the “parenting” role. This may satisfies fundamental psychological needs such as autonomy, competence, and relatedness, thereby being linked to reproductive motivation. This intrinsic motivation transforms into childbearing from a social norm, a self-driven choice for personal reasons. Ultimately boosting fertility intentions through this motivational shift. Finally, the positive emotional experiences derived from pet ownership and the resulting boost in confidence regarding family life are associated with significantly reduce individuals’ sensitivity to reproductive pressure. Pet companionship and interaction provide timely emotional comfort, alleviating anxiety and negative emotions. The accumulation of such positive emotions relates to enhanced individuals’ life satisfaction and happiness, which is associated with their confidence to face the challenges associated with childbearing. This, in turn, is indirectly linked to increased fertility intentions. Conversely, these emotional benefits may also reduce the perceived necessity of human children for fulfillment, particularly when structural barriers make childbearing impractical. These findings suggest that economic constraints may contribute to substitution effects between pet ownership and childbearing.

## Data Availability

The raw data supporting the conclusions of this article will be made available by the authors, without undue reservation.

## Footnotes

1
www.wjx.cn
